# Patient and nurse preferences for implementation of bedside handover: Do they agree? Findings from a discrete choice experiment

**DOI:** 10.1111/hex.12513

**Published:** 2016-11-02

**Authors:** Jennifer A. Whitty, Jean Spinks, Tracey Bucknall, Georgia Tobiano, Wendy Chaboyer

**Affiliations:** ^1^ Norwich Medical School Faculty of Medicine and Health Sciences University of East Anglia Norwich Norfolk UK; ^2^ School of Pharmacy Faculty of Health and Behavioural Sciences The University of Queensland Brisbane Qld Australia; ^3^ Centre for Applied Health Economics Menzies Health Institute Queensland Nathan Qld Australia; ^4^ Centre for Quality and Patient Safety School of Nursing and Midwifery Faculty of Health Deakin University Geelong Vic. Australia; ^5^ National Centre of Research Excellence in Nursing Interventions for Hospitalised Patients Menzies Health Institute Queensland Griffith University Gold Coast Qld Australia; ^6^ Alfred Health Melbourne Vic. Australia

**Keywords:** communication, discrete choice experiment, nursing, patient safety, preferences

## Abstract

**Objective:**

To describe and compare patients' and nurses' preferences for the implementation of bedside handover.

**Design:**

Discrete choice experiment describing handover choices using six characteristics: whether the patient is invited to participate; whether a family member/carer/friend is invited; the number of nurses present; the level of patient involvement; the information content; and privacy.

**Setting:**

Two Australian hospitals.

**Participants:**

Adult patients (n=401) and nurses (n=200) recruited from medical wards.

**Main outcome measures:**

Mean importance scores for handover characteristics estimated using mixed multinomial logit regression of the choice data.

**Results:**

Both patient and nurse participants preferred handover at the bedside rather than elsewhere (*P*<.05). Being invited to participate, supporting strong two‐way communication, having a family member/carer/friend present and having two nurses rather than the nursing team present were most important for patients. Patients being invited to participate and supporting strong two‐way communication were most important for nurses. However, contrary to patient preferences, having a family member/carer/friend present was not considered important by nurses. Further, while patients expressed a weak preference to have sensitive information handed over quietly at the bedside, nurses expressed a relatively strong preference for handover of sensitive information verbally away from the bedside.

**Conclusions:**

All participants strongly support handover at the bedside and want patients to participate although patient and nurse preferences for various aspects of bedside handover differ. An understanding of these preferences is expected to support recommendations for improving the patient hospital experience and the consistent implementation of bedside handover as a safety initiative.

## 1 Introduction

As the major contributing factor to adverse events in hospitals, miscommunication has been a target for improvement for over a decade.^1^ Notably, clinical handover is viewed as a high‐risk process because of the possibility of incomplete, inaccurate or even misleading information being communicated.^2^ Clinical handover was one of five high‐risk areas targeted in the World Health Organization's (WHO's) High Five campaign to improve patient safety.^3^ In Australia, it is recommended that bedside handover includes active patient participation;^3^, ^4^ however, this is not always realized in practice.^5^, ^6^ As a way of supporting consistent implementation of bedside handover, it is timely to investigate the preferences of patients and nurses for this important communication activity.

Internationally, there has been a resurgence of interest in bedside shift‐to‐shift nursing handover. In a recent systematic review, improved patient care, fewer patient complaints and improved patient outcomes such as fewer falls and other clinical incidents were associated with bedside handover.^7^ However, nurse preferences and expectations of handover may differ to patient preferences,^5^ potentially challenging the consistent implementation of bedside handover.^6^ For instance, researchers report nurses perceive patient confidentiality to be compromised when handover occurs at the bedside.^8^ One study reported on how nurses handled sensitive information to limit this issue,^9^ while in another study, patients did not report confidentiality concerns.^10^

This study quantifies and compares the preferences of adult medical patients and nurses for the characteristics of bedside handover. An understanding of these preferences is expected to support recommendations for improving the patient hospital experience and the implementation of bedside handover.

## 2 Methods

### 2.1 Research design

A discrete choice experiment (DCE) was used to elicit the preferences of patients in and nurses working on medical wards across two Australian hospitals. The DCE is a type of survey commonly used to measure preferences around the provision of health care.^11^ Participants are asked to make a series of choices between hypothetical scenarios: in this case, alternative bedside handover profiles. The profiles are described by a number of attributes (characteristics of handover), the levels of which are varied across the different choices. The choice data are analysed using regression modelling, which indicates the relative importance of different attribute levels in driving handover choice.

### 2.2 DCE survey

The development of the survey has been published.^12^ In the DCE, participants were asked to make six (patients) or nine (nurses) choices between two different bedside handover alternatives. A third alternative of “I would prefer handover away from my bedside” was also included in each choice set for both groups, enabling participants to “opt out” of bedside handover, if they preferred. Six attributes each with between two and three levels were used to describe the different bedside handover alternatives (Table 1). All participants were asked to imagine the day‐to‐evening handover. The wording for the nurse choice sets was similar to that of patients, with some minor changes in pronouns as detailed in Table 1.

**Table 1:** Attributes and levels used to describe bedside handover

Attribute	Levels
I am (The patient is) invited to participate	Yes; No
Number of nurses present at the handover	Only the nurse leaving and the nurse coming on; The nursing team leaving and the team coming on
Family member, carer or trusted friend (of the patient) allowed to be present	Yes; No
Level of (patient) involvement	I (The patient can) hear what is said; I (The patient can) hear what is said and I am (is) asked questions; I (The patient can) hear what is said, I am (is) asked questions and I can speak up at any time
What information related to your (patient) care is discussed	Information about my (the patient's) medical condition only; Information about my (the patient's) medical condition and plan for care
Confidentiality and privacy	Sensitive information is handed over quietly at my (the) bedside; Sensitive information is handed over verbally away from my bedside; Sensitive information is handed over in written form

Nurse wording reflected in brackets.

The attributes and levels were developed based on semi‐structured interviews undertaken with 20 medical patients and 20 nurses to explore their perceptions of patient participation in bedside handover^12^, ^13^ and an expert consensus group.^12^ The attribute levels were combined into bedside handover profiles using a D_p_‐efficient experimental design estimated in NGene (ChoiceMetrics Pty Ltd, Sydney, New South Wales, Australia Version 1.1.1 2012).^14^ For patients, the full design consisted of 18 choice sets, which were blocked into three survey versions, each containing six choice sets. The same 18 choice sets were used for nurses; however, for nurses, they were blocked into two survey versions, each containing nine choice sets. Nurses were considered likely to be able to respond to a greater number of choice sets without becoming burdened, which was confirmed in the pilot study.^12^ The survey versions were randomly allocated to each participant. In addition to the choice sets, the survey collected information on participant demographics, perceived health and hospital admissions (patients) or work role (nurses).

A consumer health advocate was engaged to edit the survey for plain English. The survey was piloted with 20 medical patients and 10 nurses prior to administration.^12^

### 2.3 Participants and survey administration

The survey was administered between February and June 2015. Participants were recruited from the medical wards of two tertiary referral metropolitan hospitals. Hospital 1 was a 750‐bed public hospital in the state of Queensland; whilst Hospital 2 was a 500‐bed private hospital in Victoria. At both hospitals, bedside handover was policy. A sample of 400 patients and 200 nurses (half from each hospital) was targeted, based on conventional DCE sample size guidance.^12^, ^14^

Adult medical patients (age ≥18 years) were eligible providing they had sufficient English language skills to complete the survey and had been admitted at least 2 days prior to recruitment, to ensure they had experienced bedside handover. Registered and enrolled nurses working on the same medical wards were eligible to participate. No casual nurses were recruited. Nurse unit managers or their designate initially assessed eligibility; patients and nurses were then approached and invited to participate by a researcher, who provided an information sheet, confirmed eligibility, obtained consent and administered the survey on an iPad.

### 2.4 Ethical approval

Ethics approval was obtained from the relevant hospital and university human research ethics committees. All participants provided written informed consent.

### 2.5 Data analysis

All analyses were undertaken in NLogit statistical software (ChoiceMetrics, version 5 2012). Patient and nurse data were analysed using separate mixed multinomial logit model (MMNL) regression analyses, in which the discrete choice between alternative handovers formed the dependent variable, and the attribute levels presented for each alternative were specified as independent variables to explain handover choice.

#### 2.5.1 Model specification

Analysis was undertaken using a random utility theoretical framework.^15^ The utility (satisfaction) function for handover at the bedside was specified as a linear additive equation including a constant associated with choosing handover at the bedside and the attribute levels as explanatory variables. The utility function for “I would prefer handover away from my bedside” assumed no invitation was given to the patient to participate and that a family member, friend or carer was not allowed to be present. The MMNL model was specified with the constant and all attribute levels' effects coded and assumed to be random and following a normal distribution.^16^, ^17^ Attribute levels for which the standard deviation was not significant (*P*>.05) suggesting no substantial preference heterogeneity for that attribute level were then specified to be fixed using a backward step approach.

For each attribute level, the model estimated a mean preference weight across the sample, indicating its relative importance. The extent to which preferences varied across individuals was tested by including participant characteristics in the model.^11^, ^16^ Individual characteristics were also effects coded. A backward step regression approach was used, whereby all characteristics were entered in the model, and then systematically removed with the least significant in explaining heterogeneity for any attribute level being removed first. Only those characteristics that significantly explained variation at the 5% level were retained in the final model. All preliminary models were estimated using 20 Halton draws to specify the distribution of the random coefficients; the final model was then estimated using 1000 Halton draws.^16^

#### 2.5.2 Preference scores

Scores reflecting the relative importance of different handover characteristics were derived based on the MMNL model coefficients.^18^ This was achieved for each of the patient and nurse samples by rescaling the differences between model coefficients such that the largest improvement between attribute levels was given a score of 100. All other improvements were then allocated a score of less than 100 relative to their importance for that sample.^19^ This approach allows the importance of different handover characteristics to be compared on an interval scale within (but not between) samples. That is, an importance score of 50 for a characteristic for patients suggests that characteristic is half as important for patients as a characteristic with a score of 100, and twice as important as a characteristic with a score of 25. However, this is only the case within sample (patients); a score of 50 for patients does not indicate an equal absolute importance for patients as a characteristic with a score of 50 for nurses (ie the patient and nurse scales are not identical). Nevertheless, this approach does allow a comparison of the consistency in direction and ranking of the importance of handover characteristics between patients and nurses.

## 3 Results

### 3.1 Participant characteristics

During recruitment, 1062 patients and 212 nurses were approached by the research assistants. A total of 486 patients and 205 nurses provided consent and commenced the survey, of which 401 patients and 200 nurses completed the data, giving a completion rate of 82.5% (401/486) for patients and 97.6% (200/205) for nurses.

Participant characteristics are presented in Table 2. Approximately half of patients were female, and they had been in hospital for a median of 5 days prior to survey completion. Nurses were a median age of 33 years and most (89.0%) were female. Nurses had worked in the profession for a median of 6 years and 39% had supervisory responsibility for other staff, with 11.5% being a charge nurse.

**Table 2:** Participant characteristics (Patients, N=401; Nurses N=200)

	Patients N (%) or Median (IQR)^b^	Nurses N (%) or Median (IQR)^b^
Recruited from Hospital 1	200 (49.9%)	100 (50%)
Age (years)	71.0 (IQR 57.3‐78.8)	33.0 (IQR 26.0‐46.0)
≥65 y (patients)	251 (62.6%)	
≥40 y (nurses)		74 (37.6%)
Female	216 (54.3%)	178 (89.0%)
Born in Australia	278 (69.3%)	123 (61.5%)
Aboriginal or Torres Strait Islander (ATSI) descent^a^	4 (1.0%)	2 (1%)
English mostly spoken at home	389 (97.0%)	161 (81.3%)
Has condition making it hard to verbalize with nursing staff	51 (12.7%)	–
Highest education high school or below	230 (57.4%)	–
Lives alone	108 (26.9%)	–
Previous hospital admission in the last year	266 (66.7%)	–
Overall health (1=very poor, 10=excellent)	6.0 (IQR 4.0‐8.0)	–
≥6	225 (56.1%)	–
Self‐reports any pain	184 (45.9%)	–
Length of stay at time of survey (days)	5.0 (IQR 3.0‐7.0)	–
>6 d	135 (33.7%)	–
Patients occupying other beds in room	134 (33.6%)	–
Time working as a nurse (years)	–	6.2 (IQR 2.3‐13.0)
≥5 y	–	114 (57.0%)
Most often work on a medical ward	–	171 (85.5%)
Works in more than one hospital	–	12 (6.0%)
Nurse type		
Registered	–	148 (74.0%)
Enrolled	–	3 (1.5%)
Endorsed enrolled		15 (7.5%)
Charge nurse		23 (11.5%)
Other		11 (5.5%)
Supervisory responsibility		78 (39.0%)
Number of patients in care this shift		5.0 (IQR 4.0‐6.0)

IQR, Interquartile range.

a It was not possible to test ATSI in the models, due to the relatively small number of participants identifying with an indigenous background.

b Number of individuals with missing data. Patients: Age 1, Gender 3, Health 16, Pain 8, Length of stay 1, Occupied beds in room 1, Number hospital stays in 12 mo 2, Confined to bed 4. Nurses: Age 3, Gender 3, Language 2.

### 3.2 Preferences for handover at the bedside

There were 2406 choice observations available for analysis for patient participants (401 patients each responding to six choice sets) and 1800 choice observations available for nurse participants (200 nurses each responding to nine choice sets). The MMNL model results are presented in an Online Supplement (Tables S1 and S2). Both patient and nurse participants preferred handover at the bedside rather than elsewhere (Table S1 and S2, *P*<.05); however, this was more strongly the case for patients (handover at the bedside chosen for 2350 (97.7%) choice sets for patients and 1652 (91.8%) choice sets for nurses; Mann‐Whitney *U*‐test, *P*<.001). Female patients and patients with a lower educational attainment were more likely than male patients or those with higher education to want handover at the bedside (Table S1, *P*<.05). Nurse participants who were born in Australia or who most often worked on a medical ward (which accounted for most, ie 86% of nurse participants) were also more likely to prefer handover to be undertaken at the bedside (Table S2, *P*<.05).

### 3.3 Preferences for the characteristics of bedside handover

#### 3.3.1 Patient preferences

**Figure 1:**
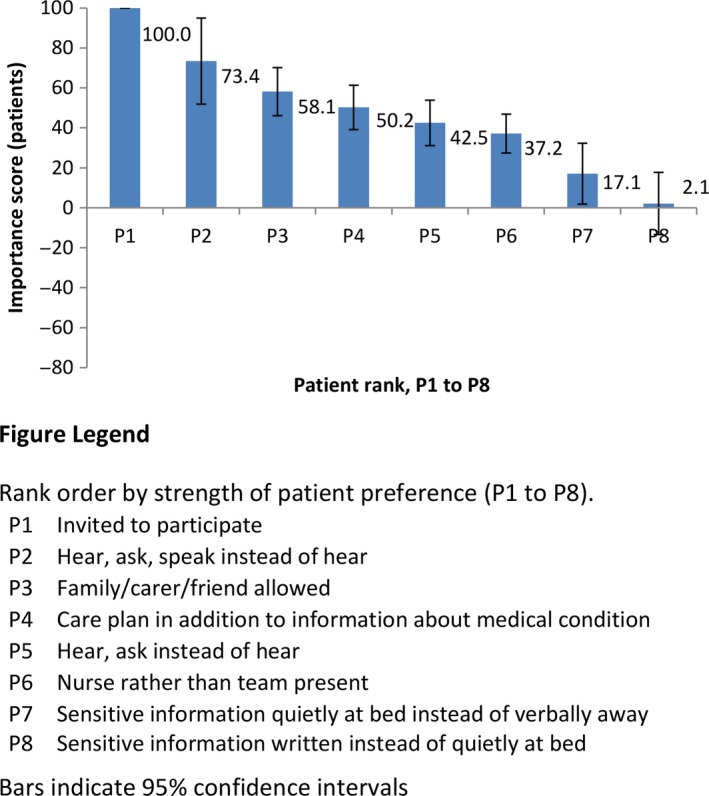
Patients: Importance scores and rank for handover characteristics [Colour figure can be viewed at wileyonlinelibrary.com]

The mean importance scores for each of the bedside handover characteristics for patients, together with their 95% confidence intervals, are presented in Table 3, and graphically in rank order of importance in Figure 1. Being invited to participate in handover (importance score 100) and being asked questions and being able to speak up as well as being able to hear what is said (2nd rank, score 73.4, 95% CI: 51.8 to 94.9) were the most important for patients. Having a family member, carer or friend able to be present (3rd rank, score 58.1; 95% CI: 46.0 to 70.2) and having a care plan in place in addition to discussing information about the patient's medical condition (4th rank, score 50.2; 95% CI: 39.1 to 61.4) were each considered to be about half as important as the patient being invited to participate in handover. Being asked questions in addition to being able to hear was ranked to be about half as important as also being able to speak (5th rank, score 42.5; 95% CI: 31.1 to 53.8). Patients preferred to have just the two nurses, one who was leaving and one who was coming on to start the shift, present rather than the whole nursing team (6th rank, score 37.2, 95% CI: 27.5 to 46.9). The handling of sensitive information was relatively unimportant; although there was a relatively small preference for having sensitive information handed over quietly at the bedside rather than away from the bedside (17.1; 95% CI: 1.9 to 32.3).

**Table 3:** Importance scores for the characteristics of bedside handover

	Patients			Nurses		
	Score	95% CI lower	95% CI upper	Score	95% CI lower	95% CI upper
Invited to participate	100.0	100.0	100.0	100.0	100.0	100.0
Hear, ask, speak instead of hear	73.4	51.8	94.9	82.4	61.0	103.8
Family/carer/friend allowed	58.1	46.0	70.2	−4.0	−14.6	6.6
Care and plan instead of care only	50.2	39.1	61.4	39.2	27.7	50.7
Hear, ask instead of hear	42.5	31.1	53.8	39.9	26.0	53.8
Nurse rather than team present	37.2	27.5	46.9	26.4	14.4	38.3
Sensitive information quietly at bed instead of verbally away	17.1	1.9	32.3	−52.2	−63.6	−40.9
Sensitive information written instead of quietly at bed	2.1	−13.4	17.6	21.5	7.2	35.8

Confidence intervals for the scores were estimated using the delta method.^39^

#### 3.3.2 Nurse preferences

**Figure 2:**
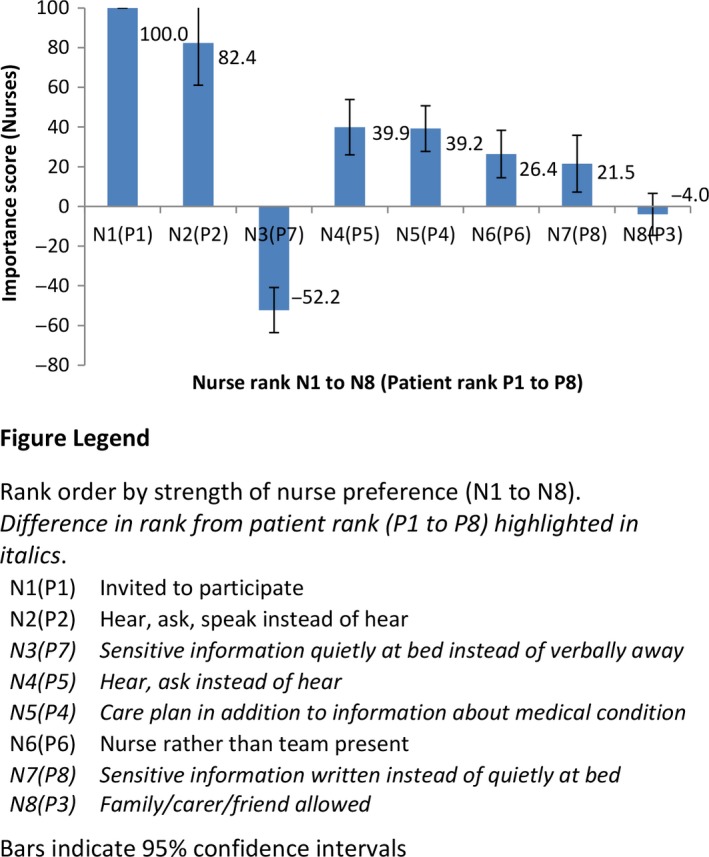
Nurses: Importance scores and rank for handover characteristics [Colour figure can be viewed at wileyonlinelibrary.com]

The mean importance scores for each of the bedside handover characteristics and 95% confidence intervals for nurses are presented in Table 3, and graphically in rank order of importance by strength of preference in Figure 2. Nurses felt that patients being invited to participate was most important (importance score 100), with the option of patients hearing, being asked question and being able to speak up ranking of similar importance (2nd rank, score 82.4; 95% CI: 61.0 to 103.8). Handing over sensitive information verbally away from the bed was strongly preferred to quietly at the bedside (3rd rank, score −52.2 for the converse; 95% CI: −40.9 to −63.6). Patients being able to hear and ask instead of hear only (4th rank, score 39.9; 95% CI: 26.0 to 53.8), having a care plan in place in addition to discussing information about the patient's medical condition (5th rank, score 39.2; 95% CI: 27.7 to 50.7) and having only the individual nurses going on/off duty rather than the whole team present (6th rank, score 26.4; 95% CI: 14.4 to 38.3) were also important for nurses. Sensitive information in written form was also preferred to quietly at the bedside (7th rank, score 21.5; 95% CI: 7.2 to 35.8). However, allowing the patient to have a family/carer/friend present was not considered to be of importance by nurses (8th rank, score −4.0; 95% CI: −14.6 to 6.6).

#### 3.3.3 Comparison of patient and nurse preferences

Patient and nurse preferences for handover can be seen to differ quite markedly. A comparison of importance scores revealed two notable differences (Figures 1 and 2). Firstly, whereas having a family member, carer or friend able to be present was the third rank of importance for patients, it was of little importance for nurses. Secondly, there were differences in preferences for handover of sensitive information. While patients expressed a relatively weak preference to have sensitive information handed over quietly at the bedside instead of verbally away from the bedside, nurses expressed a relatively strong and contradictory preference for handover of sensitive information verbally away from the bedside. Further, nurses considered handing sensitive information over in written form to be preferable to handover quietly at the bed, whilst patients did not distinguish between these options. A third difference between patient and nurse preferences is found in the switching of rank order for the importance of having a care plan (4th rank for patients, 5th for nurses) and for being asked questions as well as able to hear (5th rank for patients, 4th for nurses). However, this difference was minor and with overlapping confidence intervals.

### 3.4 Variation in preferences for the characteristics of handover across participants

Substantial variation was observed for the preferences for most characteristics of handover, even within each sample (Tables S1 and S2). For patients, four sociodemographic and health characteristics were found to be significantly associated with preference for handover characteristics; level of overall self‐reported health (*P*<.05), hospital of recruitment (*P*<.01), country of birth (*P*<.05) and level of educational attainment (*P*<.05). For nurses, four sociodemographic and work‐related characteristics were found to be significantly associated with preference for handover: hospital of recruitment (*P*<.01), country of birth (*P*<.05), ward type usually worked on (*P*<.01) and supervisory responsibility (*P*<.05).

## 4 Discussion

Patient‐centred care requires the consideration of patient preferences in care decisions.^20^ Engaging patients in care processes based on their preferences has the potential to individualize care^21^ and to improve hospital safety.^22^ The Australian Commission on Quality and Safety in Health Care has acknowledged this need to partner with consumers to promote safe care.^23^ This novel study explored the preference of patients and nurses for a critical activity that occurs in hospitals for millions of patients several times a day—the handover between nursing shifts.^24^, ^25^ We conservatively estimate that in the Australian health system alone, nursing handover occurs more than 40 million times per year (based on 54 810 public hospital beds,^26^ each having two handovers per day). Thus, handover is one of the most common safety activities to occur in hospitals, which patients can actively contribute to. The current study ranks the characteristics of handover most important for patients and for nurses and finds differences between patients and nurses. In this study, patients strongly supported handover at the bedside and both patient and nurses preferred patients to be invited to participate rather than not invited. This suggests patients and nurses value the opportunity for active patient participation in bedside handover, which is consistent with other patient and nurse views, because it promotes an opportunity for genuine patient engagement and patient‐centred care.^9^, ^27^ However, the success of patient participation in care, such as communication exchanges at care transitions, is reliant on patients being invited.^21^, ^28^ Nurses recognize it is their role to enable patient participation by encouraging and inviting patients to participate in care.^13^ The strong support expressed by nurses in the current study for patients being invited to participate suggests their sentiment supports the wide implementation of bedside handover.

Patients' and nurses' preferences emphasized the importance of two‐way communication by allowing patients to speak up and ask as well as answer questions. Mutual communication is a frequently identified way for patients to participate in nursing care.^28^, ^29^ Our findings that patients desire being updated by hearing information handed over, as well as contributing to the handover by adding information and asking questions, are largely consistent with several previous qualitative studies.^10^, ^27^, ^30^, ^31^ Nurses' value for two‐way communication during bedside handover may be attributable to their appreciation of getting the most relevant information.^32^ However, this study adds further information to our previous understanding, using a robust and systematic quantitative methodology to elicit relative patient and nurse values and ranking for different handover characteristics. There were other similarities observed in this study between patient and nurse preferences, namely both showed a preference to have just the nurse leaving and coming on duty present at handover rather than the whole nursing team. This finding has not to our knowledge been previously highlighted. Indeed, the sparse literature to date suggests nurses prefer whole team handover so they received handover on all patients.^33^ The reason for this preference in both patients and nurses needs to be explored. Speculating, patients may feel overwhelmed or disempowered by a large team being present,^34^ as the way nurses approach patients influences their confidence to participate.^5^, ^35^ Nevertheless, the joint importance of this for both patients and nurses suggests guidelines should consider implementing bedside handover in small rather than large nursing groups.

This is the first study we know of to directly compare patient and nurse preferences for handover. Despite similarities, considerable differences were found, which have important implications for bedside handover. Actual patient participation in handover appears variable with 5%‐85% of bedside handovers including patients.^6^, ^36^ Thus, the differences we found in patient and nurse preferences may explain the inconsistent enactment of bedside handover and highlight key areas to address for implementation. On the choice of who should be present at the handover, patients wanted to be permitted to have a family member, friend or carer present. This desire has been reported in previous research.^10^, ^37^ This is a key area where patient and nurse preferences differed, with nurses giving little support for allowing family members, carers or friends to be present. Researchers suggest nurses are concerned about patient privacy when handing over in front of family members; however, this concern has been easily managed by asking for patient permission prior to commencing the handover.^32^, ^33^ It is also possible that nurses may think involving families may slow the handover down.^6^, ^13^ However, these concerns can be addressed by providing patients and families guidance on the purpose of the handover and information on various other opportunities for sharing information.

Patient participants did not show a strong preference for how sensitive information was delivered, which was contrary to nurses' preferences, highlighting another area of key difference between patient and nurse preferences in our study. Nurses showed a strong preference for sensitive information to be handed over verbally away from the bedside. Nurses are concerned about breeches in privacy and confidentiality of patient information, as shown in prior studies.^6^ The findings of this DCE suggest that concerns around the handling of sensitive information may not be as strong in patients as nurses perceive them to be. Practitioners need to further understand the patient and nurse perspective around sensitive information and re‐educate nurses to develop strategies for handling sensitive information at the bedside so that nurses can feel comfortable with bedside handover without compromising patient confidentiality. These might include flexible standardized approaches to handover implementation, information brochures for patients on bedside handover, inclusion of handover practices in staff competency assessment and embedding bedside handover in the ward culture.^38^ It is also possible that the perception of what type of information is considered sensitive differs. For example, patients may perceive sensitive information to be around their personal care and body functioning, whereas nurses may perceive sensitive information to refer to prognostic information. Whilst this needs further exploration, our study suggests that clinicians can be reassured that patients are more flexible over how sensitive information is handled than they may perceive them to be.

Whilst patient and nurse preferences for handover differ, we also found variation between patients for preferred handover characteristics. Patient preference for handover was associated with gender, educational attainment, health status, country of birth and hospital of recruitment. Overall, these observations suggest bedside handover needs to be tailored to the individual patient, to accommodate variation in individual preferences. By asking patients their preference around handover (eg around handling sensitive information, the inclusion of family members and their desired extent of involvement), it would be possible with minimal cost or inconvenience to tailor handover to the choice of each individual patient. This could be achieved for example by including questions on handover preference during the patient admission process.

This study is strengthened by its inclusion of the preferences of a relatively large number of medical patients and nurses from both public and private hospitals and the high completion rate**.** Moreover, the differences between patient and nurse preferences highlighted by this study are important, and need to be considered in the development of clinical and procedural guidelines, if handover at the bedside is to be widely and consistently implemented. However, the study has not captured the views of patients with special communication needs such as those who do not speak English—their perspectives should be sought and considered separately. Further, we identified some difference in patient and nurse preference between the two hospitals included in the study—we cannot conclude the reason for these differences. For example, these differences may relate to the public/private nature of the hospitals, the hospital environment or to systematic differences in the attitudes and practices of nursing staff in different Australian states. However, these differences were only observed for characteristics related to the presence of nurse numbers or family/carer/friend at handover and were consistent between patients and nurses, likely mitigating their impact.

In summary, this study provides strong patient and nurse support for handover at the bedside. It also indicates strong support for inviting patients to actively engage in two‐way information exchanges. This may indicate that further work needs to be undertaken on the way patients are invited to participate in handover as this is strongly preferred by patients. Further, we identified areas where patient and nurse preferences differed, particularly around enabling family and friends to participate and how sensitive information is handed over. An understanding of these factors and their consideration in the development of frameworks guiding the process and design of bedside handover can be expected to improve the implementation of this important patient‐centred safety initiative in hospitals, such that it is most acceptable to patients and more likely to be implemented by nurses.

## Competing Interests

JS, WC, TB, GT, JW have nothing to declare.

## Authors' Contributions

WC, JW and TB conceived the study. JS developed the survey with contribution from all authors. JS and GT contributed to the acquisition of data. JW led data analysis and interpretation and drafted the manuscript. JS, TB, GT and WC contributed to interpretation of the data and revision of the draft for important intellectual content. JW acts as guarantor for this manuscript. All authors approved the final version and agreed to be accountable for all aspects of the work in ensuring that questions related to the accuracy or integrity of any part of the work are appropriately investigated and resolved.
