# Supplementary material for: Patient and nurse preferences for implementation of bedside handover: Do they agree? Findings from a discrete choice experiment
Source: Health Expect. 2016 Nov 2;20(4):742–50. doi: 10.1111/hex.12513 (PMC5512991; doi:10.1111/hex.12513)
Supplement: Supplementary file 1 [file HEX-20-742-s001.pdf]

S1: Patients: Preference model results (Mixed multinomial logit model MMNL)

Attribute	Level	Beta	SE	P-value	95%CI lower	95%CI upper
Handover (constant)		** 41.313	19.055	0.030	3.965	78.660
Invited to participate	Yes	*** 0.556	0.079	<0.001	0.401	0.711
	No	-0.556				
Nurses present	Nurse only	*** 0.207	0.054	<0.001	0.101	0.312
	Team	-0.207				
Family/carer/friend	Yes	*** 0.323	0.064	<0.001	0.197	0.449
	No	-0.323				
Level of involvement	Hear, ask, speak	*** 0.387	0.107	<0.001	0.176	0.597
	Hear, ask	0.043	0.060	0.476	-0.075	0.161
	Hear	-0.429				
Information	Care and plan	*** 0.279	0.064	<0.001	0.153	0.405
	Care only	-0.279				
Sensitive information	Written	0.079	0.088	0.369	-0.094	0.252
	Verbally away	-0.134	0.088	0.125	-0.306	0.038
	Quietly at bed	0.055				
**Heterogeneity around mean (for RPs)**						
	Handover: Female	** 3.535	1.737	0.042	0.131	6.939
	Handover: Good health	3.030	2.640	0.251	-2.144	8.205
	Handover: Hospital 1	-0.089	1.790	0.960	-3.598	3.420
	Handover: Born Australia	-6.549	4.314	0.129	-15.005	1.906
	Handover:	** 4.708	1.840	0.011	1.102	8.314

	Education high school					
	Invited: Female	0.040	0.061	0.509	-0.079	0.160
	Invited: Good health	0.032	0.063	0.612	-0.091	0.155
	Invited: Hospital 1	-0.102	0.063	0.108	-0.226	0.022
	Invited: Born Australia	-0.026	0.066	0.699	-0.156	0.104
	Invited: Education high school	** -0.128	0.063	0.041	-0.250	-0.005
	Nurse only: Female	0.060	0.048	0.207	-0.033	0.154
	Nurse only: Good health	** 0.103	0.051	0.043	0.003	0.202
	Nurse only: Hospital 1	***-0.308	0.056	<0.001	-0.419	-0.198
	Nurse only: Born Australia	-0.008	0.051	0.879	-0.107	0.092
	Nurse only: Education high school	0.078	0.049	0.112	-0.018	0.175
	Family/carer/friend: Female	-0.061	0.057	0.281	-0.172	0.050
	Family/carer/friend: Good health	0.053	0.059	0.367	-0.062	0.168
	Family/carer/friend: Hospital 1	***-0.239	0.061	<0.001	-0.359	-0.119
	Family/carer/friend: Born Australia	-0.040	0.061	0.509	-0.159	0.079
	Family/carer/friend:	-0.019	0.058	0.748	-0.132	0.095

	Education high school					
	Hear, ask, speak: Female	0.113	0.088	0.201	-0.060	0.286
	Hear, ask, speak: Good health	0.091	0.093	0.329	-0.092	0.274
	Hear, ask, speak: Hospital 1	0.022	0.092	0.814	-0.159	0.203
	Hear, ask, speak: Born Australia	0.136	0.094	0.145	-0.047	0.320
	Hear, ask, speak: Education high school	0.087	0.090	0.335	-0.090	0.264
	Care and plan: Female	-0.007	0.056	0.900	-0.116	0.102
	Care and plan: Good health	** 0.138	0.059	0.019	0.023	0.253
	Care and plan: Hospital 1	0.086	0.058	0.139	-0.028	0.201
	Care and plan: Born Australia	**-0.148	0.061	0.015	-0.268	-0.028
	Care and plan: Education high school	0.004	0.058	0.950	-0.109	0.116
	Written: Female	-0.073	0.078	0.354	-0.226	0.081
	Written: Good health	-0.026	0.082	0.752	-0.186	0.134
	Written: Hospital 1	0.034	0.081	0.677	-0.125	0.192
	Written: Born	-0.099	0.087	0.253	-0.269	0.071

	Australia					
	Written: Education high school	-0.004	0.080	0.956	-0.162	0.153
	Verbally away: Female	0.109	0.078	0.163	-0.044	0.263
	Verbally away: Good health	-0.031	0.082	0.703	-0.191	0.129
	Verbally away: Hospital 1	-0.048	0.082	0.555	-0.209	0.112
	Verbally away: Born Australia	0.038	0.085	0.657	-0.129	0.205
	Verbally away: Education high school	-0.002	0.080	0.976	-0.159	0.154
**Standard deviation (for RPs)**						
	Handover (constant)	** 19.556	8.805	0.026	2.298	36.813
	Invited to participate (yes)	*** 0.795	0.092	<0.001	0.615	0.975
	Nurses present (nurse only)	*** 0.362	0.096	<0.001	0.174	0.549
	Family/carer/friend (yes)	*** 0.403	0.108	<0.001	0.192	0.614
	Level of involvement (hear, ask, speak)	*** 1.017	0.129	<0.001	0.763	1.270
	Information (care and plan)	*** 0.508	0.090	<0.001	0.331	0.685

	Sensitive information (written)	*** 0.526	0.139	<0.001	0.253	0.800
	Sensitive information (verbally away)	0.225	0.176	0.201	-0.120	0.570

Note: ***, **, * ==> Significance at 1%, 5%, 10% level. RP Random Parameter
