# Supplementary material for: Patient and nurse preferences for implementation of bedside handover: Do they agree? Findings from a discrete choice experiment
Source: Health Expect. 2016 Nov 2;20(4):742–50. doi: 10.1111/hex.12513 (PMC5512991; doi:10.1111/hex.12513)
Supplement: Supplementary file 2 [file HEX-20-742-s002.pdf]

## S2: Nurses: Preference model results (Mixed multinomial logit model, MMNL)

| Attribute | Level | Beta | SE | P-value | 95%CI lower | 95%CI upper |
|---|---|---|---|---|---|---|
| Handover (constant) | | ***8.975 | 2.072 | <0.001 | 4.913 | 13.037 |
| Invited to participate | Yes | ***0.777 | 0.106 | <0.001 | 0.569 | 0.985 |
| | No | -0.777 | | | | |
| Nurses present | Nurse only | **0.205 | 0.093 | 0.028 | 0.022 | 0.387 |
| | Team | -0.205 | | | | |
| Family/carer/friend | Yes | -0.031 | 0.084 | 0.711 | -0.196 | 0.133 |
| | No | 0.031 | | | | |
| Level of involvement | Hear, ask, speak | ***0.646 | 0.148 | <0.001 | 0.357 | 0.936 |
| | Hear, ask | -0.013 | 0.107 | 0.903 | -0.222 | 0.196 |
| | Hear | -0.633 | | | | |
| Information | Care and plan | ***0.305 | 0.096 | 0.002 | 0.116 | 0.493 |
| | Care only | -0.305 | | | | |
| Sensitive information | Written | -0.048 | 0.112 | 0.670 | -0.268 | 0.172 |
| | Verbally away | ***0.430 | 0.076 | <0.001 | 0.280 | 0.579 |
| | Quietly at bed | -0.382 | | | | |
| **Heterogeneity around mean (for RPs)** | | | | | | |
| | Handover: Hospital 1 | 0.374 | 0.475 | 0.431 | -0.557 | 1.304 |
| | Handover: Born Australia | **1.456 | 0.608 | 0.017 | 0.265 | 2.647 |
| | Handover: Medical ward | **1.825 | 0.789 | 0.021 | 0.280 | 3.371 |
| | Handover: Supervisor | *-1.112 | 0.591 | 0.060 | -2.271 | 0.047 |

| | | | | | | |
|---|---|---|---|---|---|---|
| | Invited: Hospital 1 | 0.033 | 0.062 | 0.591 | -0.088 | 0.154 |
| | Invited: Born Australia | *-0.108 | 0.064 | 0.094 | -0.234 | 0.018 |
| | Invited: Medical ward | ***-0.262 | 0.092 | 0.005 | -0.443 | -0.081 |
| | Invited: Supervisor | -0.042 | 0.062 | 0.500 | -0.163 | 0.080 |
| | Nurse only: Hospital 1 | ***-0.225 | 0.061 | <0.001 | -0.344 | -0.106 |
| | Nurse only: Born Australia | -0.031 | 0.061 | 0.608 | -0.150 | 0.088 |
| | Nurse only: Medical ward | -0.044 | 0.089 | 0.621 | -0.218 | 0.130 |
| | Nurse only: Supervisor | 0.017 | 0.059 | 0.770 | -0.098 | 0.133 |
| | Family/carer/friend: Hospital 1 | 0.027 | 0.052 | 0.612 | -0.076 | 0.129 |
| | Family/carer/friend: Born Australia | 0.015 | 0.055 | 0.785 | -0.093 | 0.123 |
| | Family/carer/friend: Medical ward | 0.075 | 0.082 | 0.360 | -0.085 | 0.235 |
| | Family/carer/friend: Supervisor | 0.034 | 0.052 | 0.515 | -0.068 | 0.136 |
| | Hear, ask, speak: Hospital 1 | -0.098 | 0.087 | 0.261 | -0.268 | 0.073 |
| | Hear, ask, speak: Born Australia | 0.042 | 0.091 | 0.647 | -0.137 | 0.220 |
| | Hear, ask, speak: Medical ward | **-0.306 | 0.135 | 0.024 | -0.571 | -0.040 |
| | Hear, ask, speak: | **0.196 | 0.088 | 0.025 | 0.025 | 0.368 |

| | | | | | | |
|---|---|---|---|---|---|---|
| | Supervisor | | | | | |
| | Hear, ask: Hospital 1 | 0.045 | 0.068 | 0.509 | -0.088 | 0.177 |
| | Hear, ask: Born Australia | -0.069 | 0.069 | 0.317 | -0.205 | 0.067 |
| | Hear, ask: Medical ward | 0.159 | 0.104 | 0.125 | -0.044 | 0.362 |
| | Hear, ask: Supervisor | *-0.117 | 0.069 | 0.093 | -0.253 | 0.019 |
| | Care and plan: Hospital 1 | 0.048 | 0.057 | 0.402 | -0.064 | 0.160 |
| | Care and plan: Born Australia | -0.037 | 0.060 | 0.539 | -0.154 | 0.081 |
| | Care and plan: Medical ward | -0.002 | 0.090 | 0.979 | -0.179 | 0.174 |
| | Care and plan: Supervisor | 0.013 | 0.057 | 0.814 | -0.098 | 0.125 |
| | Written: Hospital 1 | -0.032 | 0.066 | 0.634 | -0.161 | 0.098 |
| | Written: Born Australia | **0.148 | 0.070 | 0.034 | 0.011 | 0.285 |
| | Written: Medical ward | *-0.187 | 0.105 | 0.075 | -0.392 | 0.019 |
| | Written: Supervisor | 0.053 | 0.066 | 0.422 | -0.076 | 0.182 |
| **Standard deviation (for RPs)** | | | | | | |
| | Handover (constant) | ***8.909 | 1.843 | <0.001 | 5.296 | 12.522 |
| | Invited to participate | ***0.566 | 0.075 | <0.001 | 0.420 | 0.713 |

| | | | | | | |
|---|---|---|---|---|---|---|
| | Nurses present (nurse only) | ***0.561 | 0.075 | <0.001 | 0.414 | 0.707 |
| | Family/friend/carer (yes) | ***0.355 | 0.080 | <0.001 | 0.199 | 0.512 |
| | Level of involvement (hear, ask, speak) | ***0.447 | 0.113 | <0.001 | 0.226 | 0.668 |
| | Level of involvement (hear, ask) | 0.038 | 0.178 | 0.830 | -0.310 | 0.386 |
| | Information (care and plan) | ***0.398 | 0.078 | <0.001 | 0.246 | 0.550 |
| | Sensitive information (written) | ***0.361 | 0.118 | 0.002 | 0.129 | 0.593 |

Note: ***, **, * ==> Significance at 1%, 5%, 10% level. RP Random Parameter
